# Evaluation of the Effect of Topical Melatonin Application on Immediately Placed Dental Implants Using Cone Beam Computed Tomography (CBCT)

**DOI:** 10.7759/cureus.25233

**Published:** 2022-05-23

**Authors:** Sunkavilli Ravi Kiran, Niharika Bammidi, Avula Kishan Kumar, Peruri Santosh Kumar, Yudheera Karnam

**Affiliations:** 1 Periodontics, Lenora Institute of Dental Sciences, Rajahmundry, IND; 2 Periodontology, Anusri Dental Clinic, Visakhapatnam, IND; 3 Periodontology, Svastha Dental Clinic, Chittoor, IND

**Keywords:** osseointegration, cbct, bone volumetric analysis, melatonin, immediate implants

## Abstract

Aims and objective

Immediate implants provide ideal three-dimensional positioning compared to conventional implants. There may be a gap between the surface of the implant and the bone walls of the socket when it is inserted into a recently extracted alveolus. To compensate for this, various grafting materials are used. Recently, melatonin has gained significant attention because of its inhibitory action on bone resorption. So, we conducted the study to evaluate the effects of topical melatonin application on immediately placed implants.

Methods

The present study was conducted to compare the radiographic outcomes of immediately placed dental implants with and without topical application of melatonin using cone beam computed tomography (CBCT) with nine months of follow-up. A total of 14 patients were selected, of which seven were placed without the application of melatonin and seven were placed along with the application of melatonin. Within four months, all the implants were loaded functionally with permanent prostheses. Mean crestal bone levels, bone volume, and bone density are evaluated in both groups.

Results

There was no statistically significant difference in the amount of crestal bone loss near immediately inserted implants in the melatonin group as compared to the control group. In the bone volumetric analysis and bone density, the overall mean bone volume loss is less in the melatonin group. The statistical significance of the difference was determined.

Conclusions

The present study seems to support the hypothesis that the topical application of melatonin in immediately placed dental implants provides with better osseointegration and good survival rates.

## Introduction

Over the last 40 years, osseointegration has been one of the most important scientific discoveries in dentistry. To regain eating, communication, and cosmetic function and appearance after tooth loss, dental implants are a reliable therapy option to choose from. One of the many treatment options for edentulous individuals is the use of dental implants, which have been shown to be incredibly effective [1]. Other implant procedures have been devised that use implants implanted during extractions rather than the original approach (Branemark two-stage implant installation). More than 30 years ago the idea of immediate implant placement in an extraction socket was put forward [2]. Immediate implants prevent bone resorption, maintain alveolar crest width and height, and lower the risk of dehiscence or fenestrations around dental implants [2].

The shape and size of the alveolus, as well as the implant, have a significant influence on the gap that exists between the implant surface and the socket bone walls. It was shown that implants with a rough surface may mend bone and Osseo when there is a horizontal deficiency of 2 mm or less, according to Paolantonio and coworkers vertical defects of more than 2 mm cannot be repaired by bone [3]. The result is that bone regeneration (GBR) using autografts, allografts, or alloplastic collagen plugs or combination therapy seems to have favorable clinical effects [4]. Recent research has focused on using melatonin hormone as an alternative graft material [5]. Although melatonin is a hormone, it does not work on just one organ and has various functions, such as increasing the formation of type I collagen fibers and detoxifying free radicals, which inhibit bone desorption by interfering with the activity of osteoclasts [5].

As a result, we used cone beam computed tomography (CBCT) to evaluate the effect of melatonin on peri-implant crestal bone levels, bone density, and bone volume in immediately placed dental implants. CBCT improves diagnostic accuracy and treatment planning in many situations, including the evaluation of morphology and bone quality [6,7].

## Materials and methods

This clinical investigation was done on 14 patients who had reported to the outpatient section of the Department of Periodontics, Government Dentistry College and Hospital, Vijayawada, from June 2017 to November 2018, with the approval of the hospital ethics committee, with IRB number ref 31-12-2016, and the results were published. These are divided into two groups, i.e., the control group (group A includes seven patients who received the implant fixture surrounded by Sybograft plus material and Periocol membrane) and the study group (group B includes seven patients who received the implant fixture surrounded by Sybograft plus bone graft with 3 mg melatonin powder and Periocol membrane). Those between the ages of 20 and 50 who are in excellent general health, have acceptable bone quality and quantity, good dental hygiene, and no known systemic health issues or a history of grinding or clenching are eligible to participate in the research with informed permission.

Every patient's medical history and physical examination were thoroughly investigated. In order to get an idea of how much bone space is available, a cast is made of the patient's teeth and a ridge mapping procedure is done on it. Quality and quantity of bone and localization of anatomical structures are evaluated radiographically (intraoral periapical (IOPA), orthopantomograph (OPG), CBCT).

Surgical procedure

A local anesthetic was used for all surgical operations. During this research, bone-level titanium implants with diameters of 3.75 mm, 4.2 mm, and 5 mm and lengths of 8 mm, 10 mm, 11.5 mm, and 13 mm were employed. It was necessary to luxate teeth using peristomes before they could be properly removed with extraction forceps. The granulation tissue was carefully debrided from the socket and saline solution irrigated. After removing a tooth, a periodontal probe would be used to assess the health of the socket walls, and if they were sound, the surgeon would continue with implant implantation. Repeated drilling was used to provide the foundation for the implants (2 mm, 2.8 mm, 3.5 mm, and 4.5 mm). Finally, a torque hand ratchet wrench was used to secure the final implant placement. It was grafted using hydroxyapatite (HA and β-tricalcium phosphate (TCP)) and a resorbable periodontal collagen membrane (Periocol) in control groups to fill the space between the implant shoulder and the socket's interior walls. Three milligrams of melatonin (bulk supplements) are used in the research to fill the remaining gap, and this is measured using an ESSAE FB200 Precision Weighing Balance (Essae-Teraoka Pvt. Ltd., Bangalore, India), which is then combined with HA and beta-TCP in saline and coated with a resorbable collagen membrane. An approximate boundary was created by advancing the flap coronally and then reattaching it to the skin using 3-0 mersilk sutures. It was decided to perform a CBCT scan as soon as possible following the extraction in order to check the facial socket wall's integrity. Prescribed medicines and postoperative instructions are provided. To remove stitches, a follow-up appointment was set for one week following surgery. A follow-up appointment was then planned for one, four, six, and nine months following surgery. A full month after surgery, all implants were ready for use. Four months later, the surgical cover screw was uncovered and removed under local anesthetic, and a healing cap was applied for two weeks to protect the wound. This imprint was utilized to manufacture a functional cast of the final porcelain fused to metal repair, which was subsequently bonded onto the abutment.

Indexes for plaque and sulcus bleeding (Mombelli et al.) [8] were calculated at postoperative intervals of four, six, and nine months. This soft tissue evaluation is done using a graduated plastic periodontal probe.

We were able to assess radiographic bone loss surrounding the implant using cone beam computed tomography and two reference points; the implant-bone contact point and the apical end of the implant. During the procedure, the implant's mesial and distal surfaces were tested for bone density. On their Classic® i-CAT® system, Imaging Sciences International® in Hatfield, PA uses an amorphous silicon flat-panel detector and the following set of parameters. Twenty seconds of scanning at 120 kVp and 5 mA with a 16 cm (width) × 13 cm (height) field of view (FOV) (height). "Measurement mode" was activated in the CS 3D Imaging Software (Carestream Dental India, Mumbai, India), and a distance was recorded between the two reference sites. This procedure was repeated nine months following implant implantation, and the bone loss was determined each time. Cone beam computed tomography was used to take volumetric radiographic measures surrounding the implant, with reference points being the cementoenamel junction of neighboring teeth, the root apex of neighboring teeth, and the apex of the implant itself. Bone loss surrounding an implant may be assessed using INVIVO 5.3 Software's Volumetric Analysis Tool (Anatomage, Inc., Santa Clara, CA). With the use of CS 3D Imaging Software, the density of the bone around the implant could be measured using four different reference points: the apex, the midpoint of the implant's length, and the cervical region, respectively. The values were measured 2 mm away from the implant using a 1 mm spot diameter [9].

There are paired t-tests and ANOVA tests used to compare the means of variables between baseline and nine months in the control and test groups using SPSS Version 22 Statistical Analysis Software (SPSS) (IBM SPSS Statistics for Windows, Armonk, NY, USA).

## Results

Cone beam computed tomography (CBCT) was used to examine the radiographic results of immediately inserted dental implants with and without topical melatonin treatment over the nine-month follow-up period. Table 1 provides demographic information, such as the average age and gender.

**Table 1:** Demographic details.

	Group A		Group B	
S. no	Age in years	Sex	Age in years	Sex
1	34	F	31	M
2	25	M	27	M
3	26	M	28	M
4	25	F	32	F
5	25	M	28	M
6	43	F	29	M
7	25	M	29	M
Mean	29.00+6.48		29.14+1.77	

The sulcus bleeding index had a statistically significant mean value of 0 in the fourth month, 0.43 and 0.53 in the sixth month, and 0.57 and 0.52 in the ninth month in group A. This group's sulcus draining file shows no truly large differences in mean benefits at the fourth, sixth, and tenth months. Plaque file values in group A in the fourth, sixth, and ninth months show a truly large disparity. At the fourth, sixth, and tenth months of the study, the plaque file values in group B are wildly different from those in group A. Table 2 shows the soft tissue metrics for both groups.

**Table 2:** Soft tissue parameters. *Statistically significant.

Parameter	Groups	Time	Mean	SD	F-value	P-value
Modified sulcular bleeding index	Group A	Four months	0.00	0.00	7.00	0.03*
		Six months	0.43	0.53		
		Nine months	0.57	0.52		
	Group B	Four months	0.00	0.00	3.95	0.09
		Six months	0.29	0.49		
		Nine months	0.43	0.53		
Modified plaque index	Group A	Four months	0.29	0.49	50.7	<0.01*
		Six months	0.71	0.49		
		Nine months	0.86	0.38		
	Group B	Four months	0.14	0.38	8.0	0.03*
		Six months	0.43	0.53		
		Nine months	0.57	0.53		

The mean value of crestal bone loss in group A on the mesial side is 0.80±0.15, and on the distal side is 0.70±0.22, which is statistically insignificant. The mean value of crestal bone loss in group B on the mesial side is 0.71±0.38, and on the distal side is 0.66±0.46, which is statistically insignificant. The mean value of volumetric bone levels in group A at baseline is 1.86±0.43 and after nine months is 1.33±0.38, which is statistically significant. The mean value of volumetric bone levels in group B at baseline is 2.21±0.24 and after nine months is 1.82±0.33, which is statistically significant. The mean value of bone density in group A at baseline is 302.2±6.36 and after nine months is 270.3±8.83, which is statistically significant. The mean value of volumetric bone levels in group B at baseline is 295.7±3.66 and after nine months is 279.5±4.32, which is statistically significant. The hard tissue parameters of both the groups showed in Table 3.

**Table 3:** Hard tissue parameters. *Statistically significant.

Parameter	Groups	Difference b/w baseline and nine months	Mean	SD	t-value	P-value
Crestal bone loss	Group A	Mesial	0.80	0.15	0.01	1.0
		Distal	0.70	0.22		
	Group B	Mesial	0.71	0.38	0.27	0.82
		Distal	0.66	0.46		
Volumetric bone loss	Group A	Baseline	1.86	0.43	2.92	<0.01*
		Nine months	1.33	0.38		
	Group B	Baseline	2.21	0.24	3.02	<0.01*
		Nine months	1.82	0.33		
Bone density	Group A	Baseline	302.2	6.36	14.9	<0.01*
		Nine months	270.3	8.83		
	Group B	Baseline	295.7	3.66	24.7	<0.01*
		Nine months	279.5	4.32		

## Discussion

Bone morphogenetic proteins, growth factors [10,11], and other related compounds have been utilized to increase the bone response to dental implants. Hormonal treatments that have just recently been used include growth hormones (GH), melatonin, and others. A connection has been shown between melatonin levels and bone metabolism in several research investigations [12-14]. Molecules that produce melatonin have been shown to have a direct influence on bones and adjacent cells [15-17]. Bone resorption is not prevented by rapid implant placement, and significant volume changes have been seen up to four months following immediate implantation [18,19]. As a result, the preservation of crestal bone is considered even before implant placement treatment planning [20]. Preliminary studies suggest that supplementation with melatonin may improve both the horizontal and vertical bone dimensions [21] and enhance bone density in a shorter period of time [22,23]. Melatonin was utilized in this investigation because it has been shown to reduce the generation of free radicals in osteoclasts, a kind of bone cell. Osteoclastogenesis [24] produces a free radical, which is detoxified by the antioxidant properties of vitamin E. The overall mean marginal bone loss from baseline to nine months in group A is 1.5±0.33 and in group B is 1.37±0.56, which is in accordance with the study conducted by El-Gammal et al. [25] and Hazzaa et al. [26]. Our results show that there is a reduction of crestal bone loss in group B (melatonin placed implants) than in group A (implants without melatonin). According to research done by Guardia et al. [27], melatonin functions by preventing osteoclasts from forming. As no bone remodeling occurs, this case study shows a direct action with a very limited time horizon: just the existence of inter-thread bone and the whole implant surrounding the bone area. For this reason, melatonin continues to work on a section of the bone that has undergone vigorous implant insertion, requiring remodeling by osseous matrix creation that takes five to eight weeks. Aside from that, melatonin has been shown to inhibit the formation of osteoclasts by lowering the expression levels of receptor activators of nuclear factor-kappa B ligand (RANKL) and OPG [13,14].

Low radiation dosage, quick scanning, isotropic picture quality, an adjustable field of view, and the ability to interact with data through a personal computer characterize cone beam computed tomography, a three-dimensional imaging modality [28]. The uniqueness of the present study is the volumetric analysis of peri-implant-bone levels using digital imaging and communications in medicine (DICOM) images taken from the i-CAT machine, CS 3D Imaging Software, and INVIVO 5.3 Software by using fixed reference points that remain stable throughout the study.

The mean value of volumetric bone levels in groups A and B at baseline and nine months is statistically significant. The overall mean of bone volume loss in group A is 0.53±0.23 from baseline to nine months, and in group B, the mean bone volume loss is 0.39±0.17 from baseline to nine months. These results show that there is a reduction in bone volume loss in group B (implants placed with melatonin) compared to group A (without melatonin). It is possible to stimulate osteoblastogenesis while inhibiting osteoclastogenesis via the modulation of osteoprotegerin and RANKL production and release by osteoblasts. Additionally, melatonin's antioxidant and free-radical scavenging properties assist in maintaining bone health [28].

At baseline, bone density in both the groups was similar, and at nine months, there was a statistical reduction in bone density in both the groups. However, in group B this reduction is less, with a mean difference of 16.4±1.67 at nine months of follow-up. In group A, the mean difference at nine months is 31.8±5.65. This reduction in both groups at nine months is statistically significant. This reduction in bone density could be due to normal bone remodeling at bone-implant contact and requires further long-term evaluation.

It has been shown that melatonin increases OPG expression while decreasing RANKL expression in vitro, which is the molecular mechanism by which melatonin affects bone resorption. RANKL is a powerful stimulator of osteoclastogenesis and osteoclast activity, whereas OPG is a powerful inhibitor. Inhibition of osteoclast activity would be seen by a decrease in osteoclast numbers as well as an increase in the number of osteoclasts already existing. After a period of five to nine days, melatonin increased the release of characteristics for bone sialoprotein, basic phosphatase, and osteocalcin [12]. At any point throughout the study's follow-up period, there was no indication of peri-implant radiolucency in either the group A or group B individuals studied here. According to Smith and Zarb [29], our findings are in agreement with theirs.

Since the average of the two indices was no more than 1, indicating at most moderate inflammation, soft tissue health remained generally acceptable during the evaluation time frame. Nine months later, all the implants had osseointegrated completely, with an overall success rate of 100%. Because of the limited sample size, the study's success rate was shown to be much greater than that of previous studies.

## Conclusions

A comparison of implant placements with and without melatonin treatment showed improved marginal bone levels and peri-implant health in the former group. However, when compared to the control, these values are statistically insignificant. Neither group had a single failure rate. Correct patient selection, aseptic surgical technique, non-traumatic extraction with preservation of the buccal cortical plate, and the use of biological mediators like melatonin as topical agents are all possible reasons for the observed results.

The findings of this research seem to support the concept that the administration of melatonin to immediately inserted dental implants improves osseointegration and increases survival rates. Due to the study's limitations, which include the limited number of patients evaluated and the short follow-up period following their treatment, a larger sample of patients with a longer follow-up time is necessary to confirm the results.
